# Do Beta-Blockers Really Matter in Patients with Myocardial Infarction Without Left Ventricular Systolic Dysfunction? Comment on Sabina et al. Beta-Blockers in Patients with Myocardial Infarction and Preserved Left Ventricular Ejection: A Systematic Review and Meta-Analysis of Randomized Controlled Trials. *J. Clin. Med*. 2025, *14*, 150

**DOI:** 10.3390/jcm14072247

**Published:** 2025-03-26

**Authors:** Vincenzo Acerbo, Arturo Cesaro, Paolo Calabrò

**Affiliations:** 1 Department of Translational Medical Sciences, University of Campania “Luigi Vanvitelli”, 80131 Naples, Italy; vincenzoacerbo1@gmail.com (V.A.); paolo.calabro@unicampania.it (P.C.); 2 Division of Cardiology, A.O.R.N. “Sant’Anna e San Sebastiano”, 81100 Caserta, Italy

Beta-blockers represent a key treatment pillar for reducing mortality and morbidity in patients with myocardial infarction (MI) and stable heart failure with a reduced ejection fraction (HFrEF). At the same time, its prognostic relevance is still uncertain in the setting of MI with a mildly reduced (mrEF) or preserved EF (pEF) [1]. The recommendation for beta-blockers as a cornerstone of secondary prevention after MI derives from trials performed in the pre-reperfusion era, before the contemporary high-sensitivity cardiac troponins-based diagnosis of MI and its treatment with percutaneous coronary intervention (PCI), antithrombotic drugs, lipid-lowering therapies, and renin–angiotensin–aldosterone system (RAAS) antagonists; these advances in myocardial reperfusion and pharmacotherapy have questioned the role of life-long beta-blockers in patients with MI without left ventricular dysfunction and no other primary indication for beta-blocker therapy.

Current US guidelines provide clear instructions for secondary prevention after MI, claiming that there is no cardiovascular benefit in the routine use of beta-blockers in stable MI patients with a preserved EF (class III, level of evidence C) and suggesting that the indication for the long-term (>1 year) use of beta-blockers should be re-assessed in patients with chronic coronary syndrome who were started on beta-blocker therapy for previous MI in the absence of an LVEF ≤ 50%, angina, uncontrolled hypertension, or arrhythmias (class IIb, level of evidence B). These recommendations support the clinical rationale that beta-blockers may be unnecessary for the further reduction of residual cardiovascular risk in MI patients with a normal LVEF after complete revascularization in the presence of guideline-directed medical therapy (GDMT) with antiplatelet drugs, statins, and angiotensin-converting enzyme inhibitors or angiotensin-receptor blockers [2,3].

In contrast, the European guidelines propose a more generic clinical approach, stating that the routine use of beta-blockers should be considered for all patients with acute coronary syndrome (ACS), irrespective of their LVEF (class IIa, level of evidence B), and that the long-term benefit of beta-blocker therapy in patients with MI without a reduced EF remains a significant gap in our knowledge that should be further clarified [4,5]. Of note, apart from the small CAPITAL-RCT (Carvedilol Post-Intervention Long-Term Administration in Large-scale Randomized Controlled Trial), which found no additional cardiovascular benefit of long-term carvedilol therapy in 801 Japanese patients with an LVEF ≥ 40% after an uncomplicated STEMI (ST-segment elevation myocardial infarction) and successful PCI, the referenced studies from the reperfusion era behind the latest clinical practice guidelines and recommendations for beta-blockers in MI patients without left ventricular dysfunction are non-randomized trials, observational studies, or registries of beta-blockers in patients after revascularization, providing conflicting results and a lack of robust evidence [6].

The recently published article by Sabina et al., entitled “Beta-Blockers in Patients with Myocardial Infarction and Preserved Left Ventricular Ejection: A Systematic Review and Meta-Analysis of Randomized Controlled Trials” sheds light on this critical and controversial topic [7]. 

Their systematic review and meta-analysis include three RCTs, namely the CAPITAL-RCT study and two recent European open-label trials, the REDUCE-AMI (Randomized Evaluation of Decreased Usage of Beta-Blockers after Acute Myocardial Infarction) and the ABYSS (Assessment of Beta-Blocker Interruption 1 Year after an Uncomplicated Myocardial Infarction on Safety and Symptomatic Cardiac Events Requiring Hospitalization), encompassing a total of 9242 MI patients with an LVEF ≥ 40% at the time of revascularization [8,9]. The authors found that beta-blocker therapy (mostly Metoprolol, Bisoprolol, Carvedilol, Acebutalol, Atenolol, and Nebivolol) was ineffective at reducing mortality, recurrent MI, and stroke compared to no beta-blocker therapy over a follow-up period of 3 to nearly 4 years.

Beta-blockers, by antagonizing sympathetic beta-adrenergic receptors, prevent the cardiotoxic effect of catecholamines and exert several cardiovascular effects: (I) an anti-ischemic effect by decreasing myocardial oxygen demand and increasing myocardial perfusion through reducing the heart rate, systolic blood pressure, and cardiac contractility; (II) an anti-arrhythmic effect by decreasing spontaneous atrial or ventricular ectopic electrical spikes, slowing atrio-ventricular (AV) conduction, and increasing the refractory period of the AV node; and (III) an anti-remodeling effect on the left ventricle by attenuating cardiac beta-adrenergic receptor downregulation, decreasing RAAS activation and inhibiting pathologic calcium handling in cardiomyocytes. Third-generation beta-blockers (i.e., Carvedilol, Nebivolol, and Labetalol) provide additional cardiovascular benefits: (I) a peripheral vasodilatory effect by causing both endothelium-independent vasodilation through the antagonism of alpha-adrenergic receptors and endothelium-dependent vasodilation through the stimulation of nitric oxide release from endothelial cells via ATP efflux; (II) an anti-oxidant effect by preventing free radical damage. In general, beta-blockers are well-tolerated, but high doses of these drugs may be linked to various side effects, fatigue, cold intolerance, depression, bradycardia, AV block, peripheral vasoconstriction, postural hypotension, bronchospasm, and sexual dysfunction such as erectile dysfunction and loss of libido, masking warning symptoms of hypoglycemia (tremor, tachycardia). Although the anti-inflammatory and anti-oxidant effects of beta-blockers have also been hypothesized, no solid data exist on these additional potential effects.

Moreover, the progressive upregulation of beta-adrenergic receptors during chronic treatment predisposes patients to a dangerous rebound phenomenon in cases of the immediate withdrawal of beta-blockers, leading to heightened sympathetic activity with tachycardia, elevated blood pressure, angina episodes, and psychological impacts like anxiety or agitation [10].

Sabina et al. [7], in their comprehensive meta-analysis, resized the prognostic benefit of beta-blockers for long-term secondary prevention in MI patients with preserved left ventricular function and encouraged the deprescription of routine beta-blocker use in patients without an HFrEF, residual angina, or arrhythmias. In MI patients with a pEF, excessive sympathetic drive is less pronounced than in those with a reduced EF, potentially diminishing the necessity of beta-adrenergic antagonism and autonomic regulation. However, a few remarks should be made before jumping to hasty conclusions.

First of all, in the ABYSS trial, stopping the use of long-term beta-blockers was not found to be non-inferior to a strategy of beta-blocker continuation over a 3-year follow-up period in patients with a history of MI and an LVEF ≥ 40%; the interruption of beta-blocker treatment resulted in 10 beats-per-minute higher heart rates and 3.7/3.9 mmHg higher systolic and diastolic blood pressure and was associated with a numerical increase in hospitalizations for coronary-related reasons, specifically angina or ischemia and angiography. Moreover, Silvain et al. [9,11] also reported the absence of an improvement in quality of life measured using the European Quality of Life–5 Dimensions (EQ-5D) questionnaire after 6 months of beta-blocker discontinuation. However, the EQ-5D score is a five-question instrument mainly focused on musculoskeletal health and therefore unable to fully capture the real quality of life among patients with a mean age of 64 years.

Beta-blocker therapy has always been the standard of care in post-MI secondary prevention; however, to date, it is not possible to quantify at longer-term follow-ups the rate of post-MI patients who, while not experiencing left ventricular dysfunction or new MI, will develop coronary microvascular dysfunction or ischemia, conditions in which the clinical benefit of beta-blockers is substantial.

Therefore, especially in the context of incomplete revascularization with sub-clinical ischemia and residual coronary artery disease, not prescribing beta-blocker therapy after an MI may deprive patients of first-line anti-ischemic pharmacological protection that could be effective in avoiding inappropriate hospitalizations due to recurrent angina-like symptoms and possible exposure to a new coronary angiography; these factors negatively impact patients’ quality of life and health care system spending. So, the ABYSS results favor the continuation of a drug that is generally well tolerated and cheap. In a clinical setting where the benefit of beta-blocker treatment is uncertain, assessing its long-term safety profile is equally crucial. Of note, it is essential to specify that neither the ABYSS nor the CAPITAL-RCT includes an assessment of adverse events within the outcomes, and, to date, the REDUCE-AMI is the only study to have considered safety endpoints among its patients; although the trial was not adequately powered to address this specific issue when comparing hospitalization rates for bradycardia, advanced AV block, hypotension, syncope, pacemaker implantation, and exacerbations of asthma or COPD, no significant difference in the rate of occurrence was found between the patients stopping and continuing beta-blockers.

In RCTs, the outcomes of the long-term continuation of beta-blocker therapy have always been considered a class effect, and data specifically focused on third-generation beta-blockers, which due to their vasodilator and anti-oxidant properties could demonstrate favorable cardiovascular effects on soft endpoints (such as hospitalizations for angina and quality of life) and safety endpoints, are not yet available.

When considering MI patients with an mrEF (from 40 to 49%), doubts about beta-blocker therapy are even greater. From a pathophysiological point of view, beta-blockers may be really useful in this subgroup of patients, not only for their anti-ischemic effect but also for their anti-remodeling effect on the left ventricle. Unfortunately, as suggested by Sabina et al. [7] in their meta-analysis, for patients after MI with an mrEF, it is not possible to extrapolate robust considerations, since the ABYSS study only enrolled 338 mrEF patients, the REDUCE AMI study did not enroll mrEF patients, and for the CAPITAL study no supplementary data are available to quantify the sample sizes for each EF subgroup.

The REBOOT-CNIC (TREatment With Beta-blockers After myocardial Infarction withOut Reduced Ejection fraction [NCT03596385]), the BETAMI (BEtablocker Treatment After Acute Myocardial Infarction in Patients Without Reduced Left Ventricular Systolic Function [NCT03646357]), the DANBLOCK (Danish Trial of Beta Blocker Treatment After Myocardial Infarction Without Reduced Ejection Fraction [NCT03778554]), and the SMART-DECISION (Long-term Beta-blocker Therapy After Acute Myocardial Infarction [NCT04769362]) are four ongoing pragmatic prospective large-scale RCTs randomizing a total of more than 24,500 MI patients without left ventricular systolic dysfunction to either beta-blocker treatment or control: the REBOOT-CNIC, the BETAMI, and the DANBLOCK will also provide more substantial data in patients with MI and an mrEF; in addition, BETAMI and DANBLOCK will also address the safety profile of beta-blockers [11]. By filling in these gaps in the evidence, these studies will avoid the routine prescription of indiscriminate beta-blockade to all patients in the post-MI setting and help identify any select groups of patients who might benefit instead (Figure 1).

## Figures and Tables

**Figure 1:**
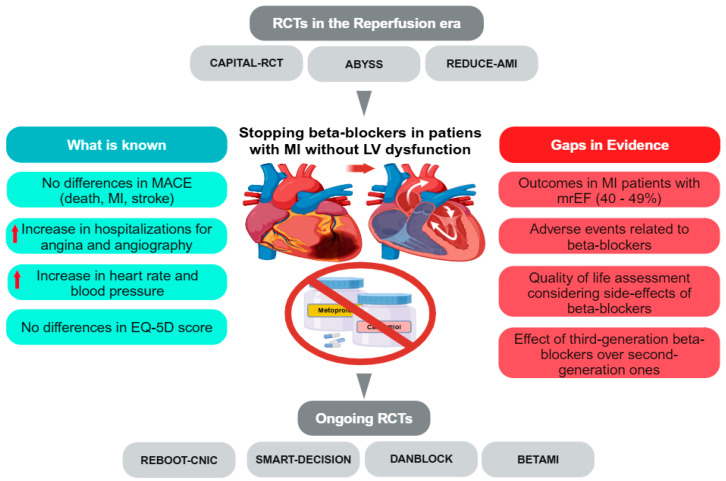
There is more doubt than certainty in stopping the use of long-term beta-blockers in patients with MI without left ventricular systolic dysfunction. RCTs: randomized controlled trials. MACE: major adverse cardiovascular event. EQ-5D: European Quality of Life–5 Dimensions. MI: myocardial infarction. mrEF: mildly reduced ejection fraction.
